# The complete chloroplast genome of *Abies ernestii* Rehder (Pinaceae) and its phylogenetic implications

**DOI:** 10.1080/23802359.2022.2109435

**Published:** 2022-08-17

**Authors:** Yi-Zhen Shao, Zhi-Yuan Shi, Zhao Wang, Wei Wang, Yun Chen, Qian Wen

**Affiliations:** a College of Life Sciences, Henan Agriculture University, Zhengzhou, China; b College of Resources and Environmental Sciences, Henan Agriculture University, Zhengzhou, China

**Keywords:** *Abies ernestii*, chloroplast genome, SSR analysis, comparative genome analysis, phylogenetic analyses

## Abstract

*Abies ernestii* Rehder is endemic to the montane regions of Southwest China. Till now, phylogenetic relationships between *A. ernestii* and other closely related species remain unclear. In this study, we first characterized the complete chloroplast (cp) genome of *A. ernestii*. The whole cp genome was 121,841 bp in size, including one hundred and thirteen genes. Results of comparative cp genome revealed that only *ycf*1 and *ycf*2 was characterized by a considerable variation. Our phylogenetic analyses supported the monophyly of the genus *Abies* and revealed a clear separation between *A. ernestii* and *A. chensiensis* Tiegh. This study highlights the significance of using cp genomes to examine species boundaries among closely related fir species.

## Introduction

*Abies ernestii* Rehder is distributed across Southwest China and characterized by high economic and ecological importance (Farjon and Rushforth 1989; Farjon 1990). It grows in cold, moist forests of the Hengduan Mountain region at 2600–3800 m elevations, with annual precipitation of 1100–2200 mm (Kuan 1981). *Abies ernestii* differs from *Abies chensiensis* in the length of needles and color of cones (green or pale purplish), in addition to its distribution in the north and west of the Sichuan province, extending into extreme north-western Yunnan province (Liu 1971). However, the species delimitation between *A. ernestii* and *A. chensiensis* has been a continuous source of debate (Liu 1971; Farjon 2001).

Recently, several studies have been conducted on the species delimitation between *A*. *ernestii* and *A*. *chensiensis* using morphological or molecular data (Suyama et al. 2000; Xiang et al. 2004, 2009, 2015; Liepelt et al. 2010; Aguirre-Planter et al. 2012). However, no robust phylogenetic relationships have been reported owing to the lack of high-resolution molecular markers (e.g. complete chloroplast [cp] genomes) (Shao and Xiang 2015; Xiang et al. 2018). Therefore, several distinct taxonomic combinations have been proposed: *A. ernestii* was treated as an independent species, a variety, or a subspecies of *A. chensiensis* (Liu 1971; Shao and Xiang 2015). In addition, *A. chensiensis* and *A. ernestii* were treated as identical in the studies conducted by Handel-Mazzetti (1929) and Dallimore and Jackson (1966). Thus, a more rigorous approach using high-resolution molecular markers is required to delimitate the relationships between *A. ernestii* and *A. chensiensis*.

In this study, we first determined the cp genome of *A. ernestii* using next-generation sequencing technology. Thereafter, we compared it with other available genomes to explore their genetic divergence, develop molecular markers, and reconstruct phylogenetic relationships. This study evaluated the significant value of cp genomes to examine the species boundaries among closely related fir species.

## Materials and methods

### Sample collection and DNA extraction

The specimens and leaf materials of *A. ernestii* were collected by Xianchun Zhang from Wenchuan county, Sichuan province, China (30.55°N, 103.03°E). The voucher specimen was deposited in the herbarium of the Institute of Botany, CAS (PE) (http://pe.ibcas.ac.cn/, Qin Ban, herbarium2@ibcas.ac.cn) under the voucher number 5874. The leaf materials were first desiccated in silica gel, and the Ezup plant genomic DNA prep kit was used to extract the total DNA. The relevant cp genome sequence was submitted to the NCBI database (No. MH706707). The associated numbers were PRJNA790664 (BioProject), SRP351570 (SRA), and SAMN24219951 (Bio-Sample), respectively.

### Polymerase chain reaction amplification, sequencing, and assembly

The extracted DNA was sequenced on the Illumina HiSeq X platform with libraries in 350 bp length. The clean reads were approximately 10.2 million in 150 bp length. We aligned, assembled, and annotated the reads using CLC de novo assembler (v. beta 4.6, CLC Bio, Aarhus, Denmark), GeSeq (https://chlorobox.mpimp-golm.mpg.de/geseq.html), and tRNAscan-SE v1.3.1 (Schattner et al. 2005; Tillich et al. 2017). Few adapt-related reads were recognized in the raw reads. These reads were trimmed *N* > 10% or *Q* ≤ 5 to ensure high-quality data and then assigned to the genome sequence using Velvet (Zerbino and Birney 2008). The cp genome sequence was annotated in tRNAscan-SE and GeSeq (Schattner et al. 2005; Tillich et al. 2017). To match the gene predictions, we checked all the start/stop codons and intron/exon boundaries in Sequin 15.50 and Geneious 8.0.2 (Kearse et al. 2012; Lohse et al. 2013). Finally, we annotated the sequences by comparing them with the published genomes of *A. chensiensis* (MH706706 and MH047653).

### Repeat sequence detection and comparative analysis of cp genomes

The MISA software was used to check the simple repetitive DNA sequences (SSRs) in the cp genome of *A. ernestii*. Thereafter, we detected the long repeats using the REPuter website (https://bibiserv.cebitec.unibielefeld.de/reputer/) (Kurtz et al. 2001). The parameters used in the analysis were as follows: the hamming distance was 3, maximum computed repeats were 50 bp, and minimal repeat size was 30 bp. Using the mVista program with Shuffle-LAGAN mode, we compared the whole cp genome of *A. ernestii*, *A. chensiensis* (MH706706 and MH047653), and *Abies fargesii* Franch. (MH706716) (Frazer et al. 2004). The repeat units were set to 10, 5, 4, 3, 3, and 3 for mono-, di-, tri-, tetra-, penta-, and hexa-nucleotides, respectively.

### Phylogenetic analyses

Phylogenetic analysis was performed using the complete cp genomes. We aligned the sequences containing the hotspot mutation regions and chose the maximum-likelihood (ML) analysis as the method in this study. The analysis was performed using a rapid bootstrap analysis and 1000 rapid bootstrap search steps in the RAxML v.7.8 (Stamatakis 2014). The relevant bootstrap value under each node was obtained from FigTree v.2.2. We collected the complete plastomes of 16 fir species from the NCBI database (Figure 3, Table S1). *Juniperus squamata* Buchanan–Hamilton ex D. Don in the family Cupressaceae was used as an outgroup.

**Table 1.** List of genes encoded in *Abies ernestii* chloroplast genomes.

Groups of genes	Name of genes
Ribosomal RNAs	*rrn*16, *rrn*23, *rrn*4.5, *rrn*5
Transfer RNAs	*trn*A-UGC^a^, *trn*C-GCA, *trn*D-GUC, *trn*E-UUC, *trn*F-GAA, *trnf*M-CAU, *trn*G-GCC^a^, *trn*G-UCC^,^*trn*H-GUG, *trn*I-CAU*, *trn*I-GAU^a^, *trn*K-UUU^a^, *trn*L-CAA, *trn*L-UAA^a^, *trn*L-UAG, *trn*M-CAU, *trn*N-GUU, *trn*P-GGG*, trn*P-UGG, *trn*Q-UUG, *trn*R-ACG, *trn*R-CCG, *trn*R-UCU, *trn*S-GCU*, *trn*S-GGA, *trn*S-UGA, *trn*T-GGU*, *trn*T-UGU, *trn*V-GAC, *trn*V-UAC^a^, *trn*W-CCA, *trn*Y-GUA
Subunits of photosystem I	*psa*A, *psa*B, *psa*C, *psa*I, *psa*J, *psa*M*
Subunits of photosystem II	*psb*A, *psb*B, *psb*C, *psb*D, *psb*E, *psb*F, *psb*H, *psb*I, *psb*J, *psb*K, *psb*L, *psb*M, *psb*N, *psb*T, *psb*Z
Subunits of cytochrome b/f complex	*pet*A, *pet*B^a^, *pet*D^a^, *pet*G, *pet*L, *pet*N
Subunits of ATP synthase	*atp*A, *atp*B, *atp*E, *atp*F^a^, *atp*H, *atp*I
Proteins of large ribosomal subunit	*rpl*2^a^, *rpl*14, *rpl*16^a^, *rpl*20, *rpl*22, *rpl*23, *rpl*32, *rpl*33, *rpl*36
Proteins of small ribosomal subunit	*rps*2, *rps*3, *rps*4, *rps*7, *rps*8, *rps*11, *rps*12^b^, *rps*14, *rps*15, *rps*18, *rps*19
Large subunit of RuBisco	*rbc*L
Subunits of RNA polymerase	*rpo*A, *rpo*B, *rpo*C^a^, *rpo*C2
Conserved hypothetical chloroplast reading frames	*ycf*1, *ycf*2, *ycf*3^b^, *ycf*4, *ycf*12*
ATP-dependent protease subunit P	*clp*P
Chloroplast envelope membrane protein	*cem*A
Chlorophyll biosynthesis	*chl*B, *chl*L, *chl*N
Miscellaneous proteins	*acc*D, *ccs*A, *inf*A, *mat*K

*Genes with two copies; ^a^Genes with one intron; ^b^Genes with two introns.

## Results and discussion

### Chloroplast genome features of A. ernestii

**Figure 1.**
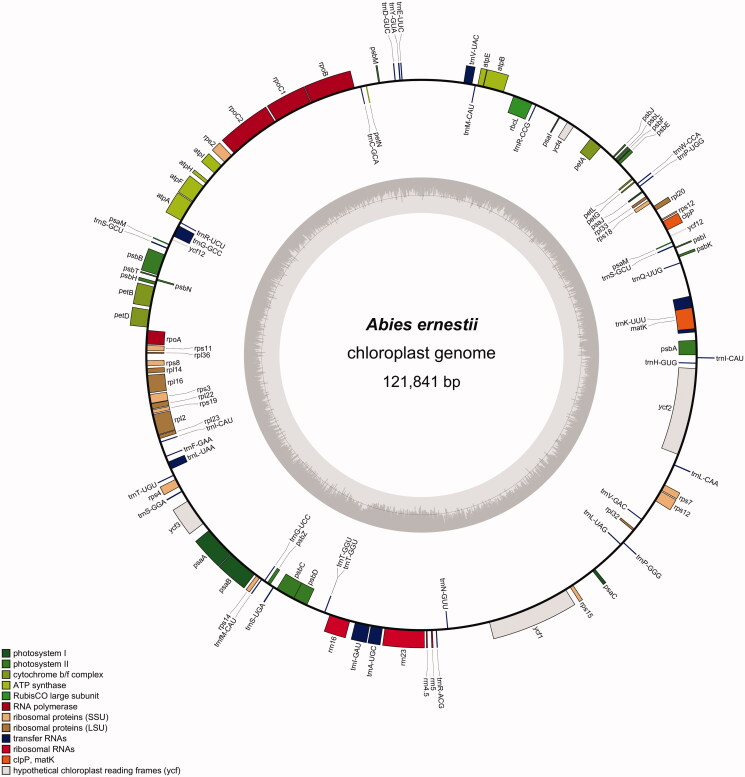
The chloroplast genome map of *Abies ernestii*. Genes shown outside the circle are transcribed clockwise, and genes inside are transcribed counter-clockwise. Genes belonging to different functional groups are color-coded. The darker grey in the inner corresponds to the GC content and the lighter grey to the AT content.

The complete cp genome of *A. ernestii* was 121,841 bp in length, and its G and C content was ∼38.30%. This genome was characterized by a typical quadripartite structure, similar to the cp genomes in Pinaceae. The large single-copy (LSC) region and short single-copy (SSC) region were 67,157 and 54,156 bp, respectively. It contained 113 genes, including peptide-encoding genes (68), tRNA genes (16), open reading frames (6), and rRNA genes (4), respectively (Figure 1, Table S2). In the LSC region, there were 53 protein-coding genes, 16 tRNA genes, and 3 open reading frames. The SSC region owned all the 4 rRNA. The IR regions were 264 bp and featured *trn*I-CAU and *trn*T-GGU. Among the encoded genes, *ycf*3 and *rpl*2 had two introns, and 11 genes (*atp*F, *trn*V-UAC, *trn*A-UGC, *trn*L-UAA, *trn*K-UUU, *trn*G-GCC, *rps*12, *pet*D, *rpo*C1, *pet*B, and *rpl*16) had one intron (Table 1). The palindromic inverted repeat, including *trn*S, *psa*M, *ycf*12, and *trn*G, was located in 52-kb inversion points and 1180 bp in length. Such inverted repeat was identical to the published cp genomes of fir species (Dong et al. 2021). In addition, all ndh genes were lost as other cp genomes of Pinaceae, such as *Keteleeria davidiana* var. *calcarean* (W. C. Cheng & L. K. Fu) Silba (Li et al. 2021). As proposed by Blazier et al. (2011), the ndh genes were initially discovered through their homology to complex I in the mitochondrial respiratory electron transport. The lack of ndh genes across various species in Pinaceae indicated its dispensability, suggesting that there might be alternative or supplementary electron transport pathways to achieve this function.

### Repeat sequence analysis

The simple sequence repeats (SSRs) have been widely used in phylogenetic and phylogeographic analyses of highly polymorphic genetic materials (Kaur et al. 2015). In this study, we detected 67 SSRs in the cp genome of *A. ernestii*, and they included 42 mononucleotide repeats, 14 dinucleotide repeats, 2 trinucleotide repeats, 7 tetranucleotide repeats, and 2 pentanucleotides repeats (Figure S1). Compared with other SSRs, the mononucleotide repeats (62.68%) were the most abundant and they contributed more to the genetic variations. Most mononucleotide repeats were A or T, and three types of dinucleotide SSRs AG/CT/AT were identified. In addition, we detected two types of trinucleotide SSRs (AAT/ATT), twelve types of tetranucleotide SSRs (AAAG/CTTT/AAAT/ATTT/AACC/GGTT/ACCT/AGGT/AGAT/ATCT/ATCC/ATGG), and four types of pentanucleotides SSRs (AACAT/ATGTT/AATCG/ATTCG) (Figure S1). Furthermore, sixty-four long repeats were revealed, and they included 37 forward repeats, 14 tandem repeats, and 13 palindromic repeats (Figure S1).

### Comparative genome analysis

**Figure 2.**
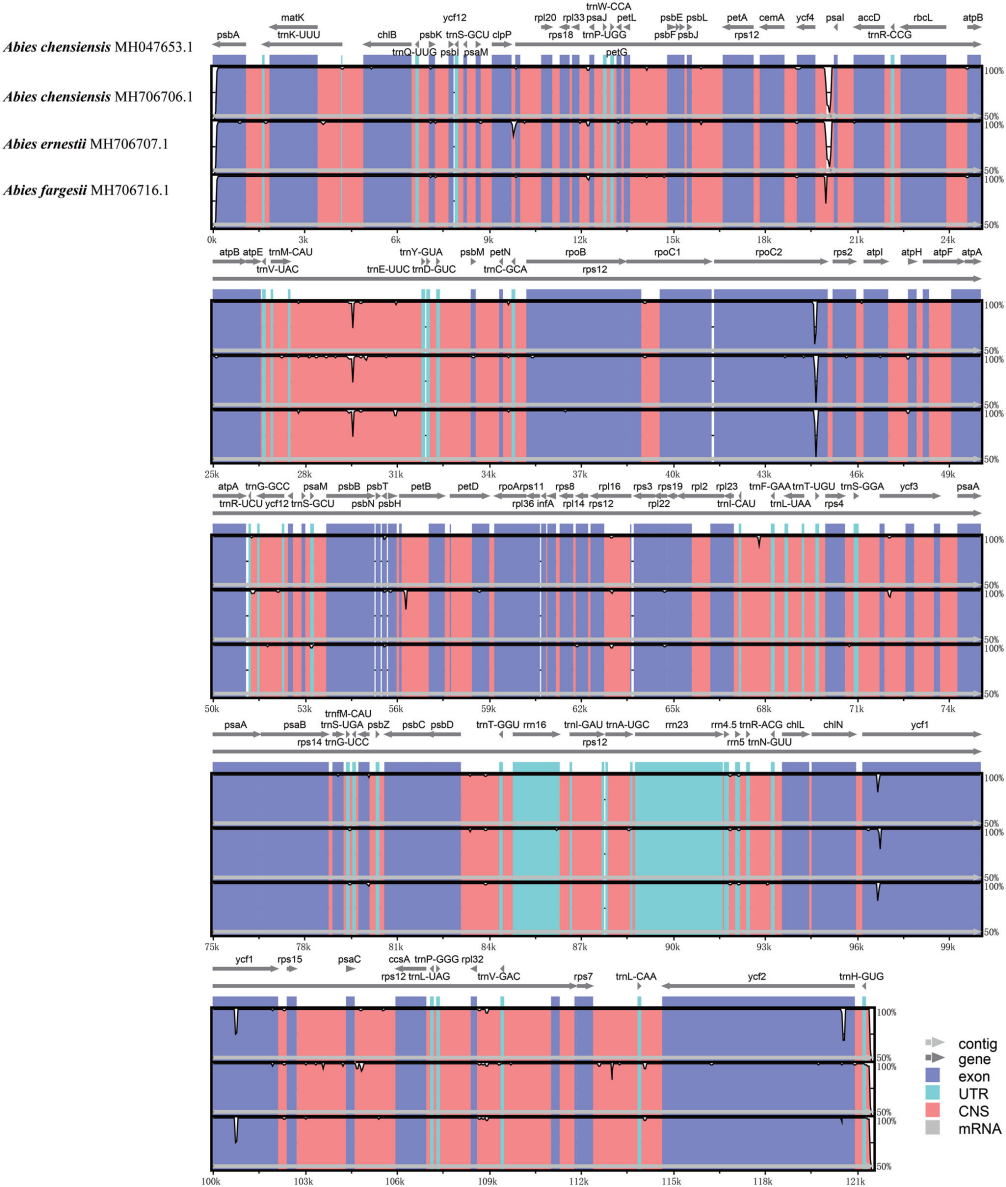
Comparison of four chloroplast genomes using the mVista alignment program, with *Abies chensiensis* (MH706706) as a reference. The *x*-axis means the midpoint of the window, and the *y*-axis means nucleotide diversity (Pi). Genome regions are color-coded as protein-coding, rRNA coding, tRNA coding, or conserved noncoding sequences.

**Figure 3.**
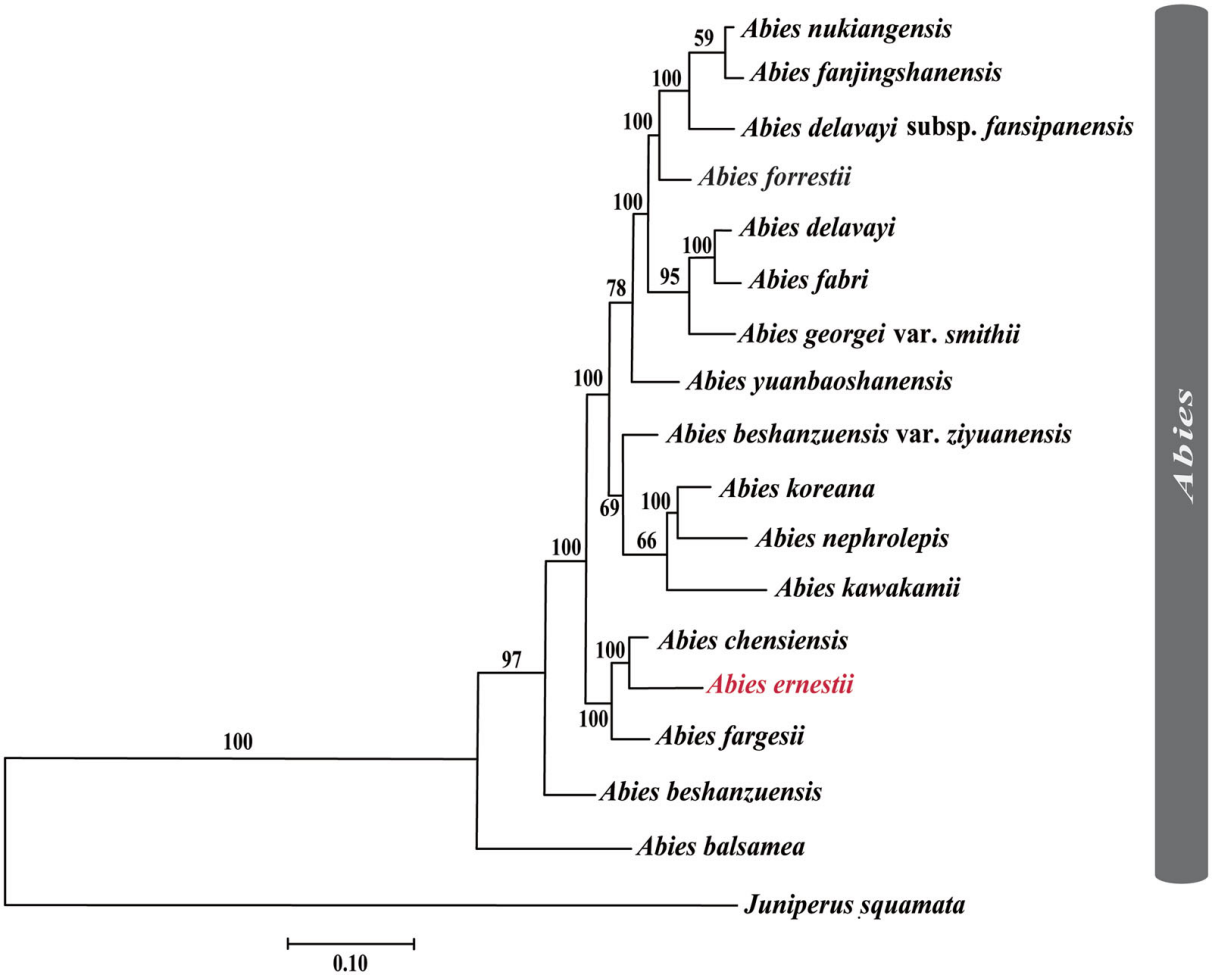
The best maximum-likelihood (ML) phylogram inferred from 17 chloroplast genomes in *Abies*, with *Juniperus squamata* as an outgroup (bootstrap values are indicated on the branches). The following sequences were used: *A. nukiangensis* MH706711 (Shao et al. 2020), *A. fanjingshanensis* MH706717 (Guo et al. 2019), *A. delavayi* subsp. *fansipanensis* MH706720 (Shao et al. 2020), *A. forrestii* MH706715 (Dong et al. 2021), *A. delavayi* MH706709 (Shao et al. 2020), *A. fabri* MH706710 (Shao et al. 2020), *A. georgei* var. *smithii* NC_054152 (Li et al. 2020), *A. yuanbaoshanensis* MH706718 (Zhang et al. 2020), *A. beshanzuensis var. ziyuanensis* MH706705 (Fu et al. 2019), *A. koreana* KP742350 (Yi et al. 2015), *A. nephrolepis* KT834974 (Yi et al. 2016), *A. kawakamii* MH706726 (Shao et al. 2019 ), *A. chensiensis* MH 706706 (Su et al. 2019), *A. fargesii* MH706716 (Guo and Xu 2019), *A. beshanzuensis* MH 476330 (Shao et al. 2018; Shao et al., 2020), *A. balsamea* MH 706725 (Wu et al. 2019), and *Juniperus squamata* MK085509 (www.ncbi.nlm.nih.gov/).

To perform the comparative genome analysis, we used three closely related firs: *A. ernestii*, *A. chensiensis*, and *A. fargesii* (Figure 2). In the phylogenetic analysis, they clustered together as a monophyletic lineage, with an extremely high bootstrap (BSML = 100) (Figure 3). Our results indicated that the IR region was more conservative than the SSC and LSC regions. Almost all the genetic variability was concentrated in the intergenic or noncoding region, corroborating the extremely low resolution among closely related fir species in previous phylogenetic studies (Figure 2) (Xiang et al. 2004, 2009, 2015). In the coding regions, only *ycf*1 and *ycf*2 were characterized by a considerable variation between *A. ernestii* and *A. chensiensis* (Figure 2). *ycf*1 had been proposed as the most variable coding region that was better than the existing plastid barcodes and could serve as a DNA barcode (Dong et al. 2015). Unfortunately, the cp markers widely used in the *Abies* phylogenetic studies, for example, *mat*K, *rpl*16, *rps*12*-rpl*20, *rps*18, *trn*C-D, *trn*S-G, and *trn*T-F, were highly conservative among the three closely related species (Xiang et al. 2018).

### Phylogenetic analysis

To infer the phylogenetic status and evolutionary relationship of *A. ernestii*, we selected 16 published cp genomes of fir species, with *Juniperus squamata* as an outgroup. Based on the best ML phylogram, 17 *Abies* species were supported as one monophyletic lineage (BS_ML_ = 100) (Figure 3). *Abies balsamea* (L.) Mill. was distributed in North America and formed a sister lineage with the firs from East Asia (BS_ML_ = 100). Among the East Asia firs, *Abies beshanzuensis* M. H. Wu was nested with others (BS_ML_ = 97). *Abies ernestii*, *A. chensiensis*, and *A. fargesii* clustered together as a monophyletic lineage, with an extremely high bootstrap (BS_ML_ = 100). Our results further indicated that *A. ernestii* and *A. chensiensis* were separated with an extremely high bootstrap (BS_ML_ = 100); thus, their current species status was well corroborated. The treatments of *A. ernestii* as a variety of *A. chensiensis*, or an identical subspecies of *A. chensiensis* were rejected (Handel-Mazzetti 1929; Dallimore and Jackson 1966). Such a robust phylogenetic relationship between *A. ernestii* and *A. chensiensis* has not been revealed previously (Xiang et al. 2004; Jaramillo-Correa et al. 2008; Semerikova et al. 2011, 2018).

This study provides new insights into the species delimitation and phylogenetic relationship in the genus *Abies*. Our results validated the reliability of using cp genome data to delimitate problematic groups at low taxonomic levels. These data provided important genetic resources for these ecologically important *Abies* species and further cp genome evolution research.

## Supplementary Material

Supplemental Material

## Data Availability

The genome sequence data supporting this study’s findings are available in GenBank of NCBI at (https://www.ncbi.nlm.nih.gov/) under accession no. MH706707. The associated BioProject, SRA, and Bio-Sample numbers are PRJNA790664, SRP351570, and SAMN24219951, respectively.
