# Supplementary material for: The complete chloroplast genome of Abies ernestii Rehder (Pinaceae) and its phylogenetic implications
Source: Mitochondrial DNA B Resour. 2022 Aug 17;7(8):1497–503. doi: 10.1080/23802359.2022.2109435 (PMC9387311; doi:10.1080/23802359.2022.2109435)
Supplement: Supplemental Material [file TMDN_A_2109435_SM2411.doc]

**Table S1.** The complete chloroplast genomes used in this study.

| **Family** | **Genus** | **Species** | **GenBank accession number** | | **Length** |
| --- | --- | --- | --- | --- | --- |
| Pinaceae | *Abies* | *Abies ernestii* Rehd. | | MH 706707* | 121841 bp |
| Pinaceae | *Abies* | *Abies chensiensis* Tiegh. | | MH 706706 | 121,795 bp |
| Pinaceae | *Abies* | *Abies nukiangensis* Cheng et L. K. Fu | | MH 706711 | 120,017 bp |
| Pinaceae | *Abies* | *Abies fanjingshanensis* W. L. Huang et al. | | MH 706717 | 120,057 bp |
| Pinaceae | *Abies* | *Abies delavayi* subsp. *fansipanensis*  (Q.P.Xiang, L.K.Fu & Nan Li) Rushforth | | MH 706720 | 120,093 bp |
| Pinaceae | *Abies* | *Abies forrestii* C. C. Rogers | | MH 706715 | 120,022 bp |
| Pinaceae | *Abies* | *Abies delavayi* Franch. | | MH 706709 | 120,141 bp |
| Pinaceae | *Abies* | *Abies fabri* (Mast.) Craib | | MH 706710 | 120,027 bp |
| Pinaceae | *Abies* | *Abies georgei* var. *smithii* (Viguie et  Gaussen) Cheng et L | | NC_054152 | 121,191 bp |
| Pinaceae | *Abies* | *Abies yuanbaoshanensis* Y. J. Lu & L. K. Fu | | MH 706718 | 121,795 bp |
| Pinaceae | *Abies* | *Abies beshanzuensis* var. *ziyuanensis* (L. K. Fu & S. L. Mo) L. K. Fu & Nan Li | | MH 706705 | 121,274 bp |
| Pinaceae | *Abies* | *Abies koreana* E. H. Wilson | | KP 742350 | 121,373 bp |
| Pinaceae | *Abies* | *Abies nephrolepis* (Trautv.) Maxim. | | KT 834974 | 121,336 bp |
| Pinaceae | *Abies* | *Abies kawakamii* (Hayata) T. Ito | | MH 706726 | 121,290 bp |
| Pinaceae | *Abies* | *Abies fargesii* Franch. | | MH 706716 | 121,799 bp |
| Pinaceae | *Abies* | *Abies beshanzuensis* M. H. Wu | | MH 476330 | 121,399 bp |
| Pinaceae | *Abies* | *Abies balsamea* (L.) Mill. | | MH 706725 | 121,574 bp |
| Cupressaceae | *Juniperus* | *Juniperus squamata* Buchanan-Hamilton ex D. Don | | MK 085509 | 127,792 bp |

*: indicated newly generated chloroplast genomes.

**Table S2.** The chloroplast genome organization of *Abies ernestii*.

| **Gene** | **Position** | **Length (bp)** | **Gene** | **Position** | **Length (bp)** | **Gene** | **Position** | **Length (bp)** |
| --- | --- | --- | --- | --- | --- | --- | --- | --- |
| *psb*A | 1 | 1062 | *psb*M | 33389 | 123 | *rps*4 | 69980 | 606 |
| *trn*K-UUU | 1609 | 2596 | *pet*N | 34336 | 90 | *trn*S-GGA | 70908 | 87 |
| *mat*K | 1855 | 1530 | *trn*C-GCA | 34747 | 71 | *ycf*3 | 71732 | 1950 |
| *chl*B | 4918 | 1545 | *rpo*B | 35213 | 3231 | *psa*A | 74285 | 2253 |
| *trn*Q-UUG | 6602 | 72 | *rpo*C1 | 38466 | 2762 | *psa*B | 76563 | 2205 |
| *psb*K | 7028 | 180 | *rpo*C2 | 41321 | 3657 | *rps*14 | 78917 | 300 |
| *psb*I | 7694 | 111 | *rps*2 | 45198 | 705 | *trn*fM-CAU | 79344 | 74 |
| *trn*S-GCU | 7880 | 88 | *atp*I | 46206 | 747 | *trn*G-UCC | 79570 | 71 |
| *psa*M | 8173 | 87 | *atp*H | 47641 | 246 | *psb*Z | 79766 | 325 |
| *ycf*12 | 8587 | 102 | *atp*F | 48137 | 1337 | *trn*S-UGA | 80330 | 87 |
| *clp*P | 9105 | 593 | *atp*A | 49517 | 1524 | *psb*C | 80622 | 1422 |
| *rps*12 | 9881 | 101943 | *trn*R-UCU | 51139 | 70 | *psb*D | 81991 | 1062 |
| *rpl*20 | 10708 | 351 | *trn*G-GCC | 51415 | 852 | *trn*T-GGU | 84367 | 72 |
| *rps*18 | 11338 | 282 | *ycf*12 | 52451 | 102 | *trn*T-GGU | 84368 | 71 |
| *rpl*33 | 11743 | 207 | *psa*M | 52880 | 87 | *rrn*16 | 84799 | 1491 |
| *psa*J | 12284 | 132 | *trn*S-GCU | 53172 | 88 | *trn*I-GAU | 86663 | 1059 |
| *trn*P-UGG | 12724 | 74 | *psb*B | 53691 | 1527 | *trn*A-UGC | 87795 | 847 |
| *trn*W-CCA | 12966 | 74 | *psb*T | 55292 | 108 | *rrn*23 | 88783 | 2807 |
| *pet*G | 13168 | 114 | *psb*N | 55481 | 132 | *rrn*4.*5* | 91694 | 103 |
| *pet*L | 13434 | 135 | *psb*H | 55692 | 228 | *rrn*5 | 92047 | 121 |
| *psb*E | 14830 | 252 | *pet*B | 56077 | 1438 | *trn*R-ACG | 92390 | 74 |
| *psb*F | 15091 | 120 | *pet*D | 57729 | 1238 | *trn*N-GUU | 93197 | 72 |
| *psb*L | 15237 | 117 | *rpo*A | 59161 | 1011 | *chl*L | 93548 | 876 |
| *psb*J | 15472 | 123 | *rps*11 | 60231 | 393 | *chl*N | 94540 | 1413 |
| *pet*A | 16646 | 960 | *rpl*36 | 60706 | 114 | *ycf*1 | 96198 | 5883 |
| *cem*A | 17832 | 786 | *inf*A | 60922 | 237 | *rps*15 | 102414 | 267 |
| *ycf*4 | 19058 | 555 | *rps*8 | 61302 | 399 | *psa*C | 104355 | 246 |
| *psa*I | 20210 | 111 | *rpl*14 | 61846 | 369 | *ccs*A | 105965 | 960 |
| *acc*D | 20867 | 969 | *rpl*16 | 62331 | 1285 | *trn*L-UAG | 107062 | 78 |
| *trn*R-CCG | 22096 | 74 | *rps*3 | 63709 | 654 | *trn*P-GGG | 107292 | 74 |
| *rbc*L | 22414 | 1428 | *rpl*22 | 64365 | 402 | *rpl*32 | 108393 | 180 |
| *atp*B | 24594 | 1479 | *rps*19 | 64806 | 279 | *trn*V-GAC | 109368 | 72 |
| *atp*E | 26081 | 414 | *rpl*2 | 65139 | 1500 | *rps*7 | 111876 | 468 |
| *trn*M-CAU | 26607 | 73 | *rpl*23 | 66659 | 276 | *trn*L-CAA | 113849 | 81 |
| *trn*V-UAC | 26873 | 617 | *trn*I-CAU | 67130 | 74 | *ycf*2 | 114656 | 6246 |
| *trn*E-UUC | 31792 | 73 | *trn*F-GAA | 68213 | 73 | *trn*H-GUG | 121227 | 75 |
| *trn*Y-GUA | 31932 | 84 | *trn*L-UAA | 68657 | 569 | *trn*I-CAU | 121614 | 74 |
| *trn*D-GUC | 32277 | 74 | *trn*T-UGU | 69643 | 73 |  |  |  |

**
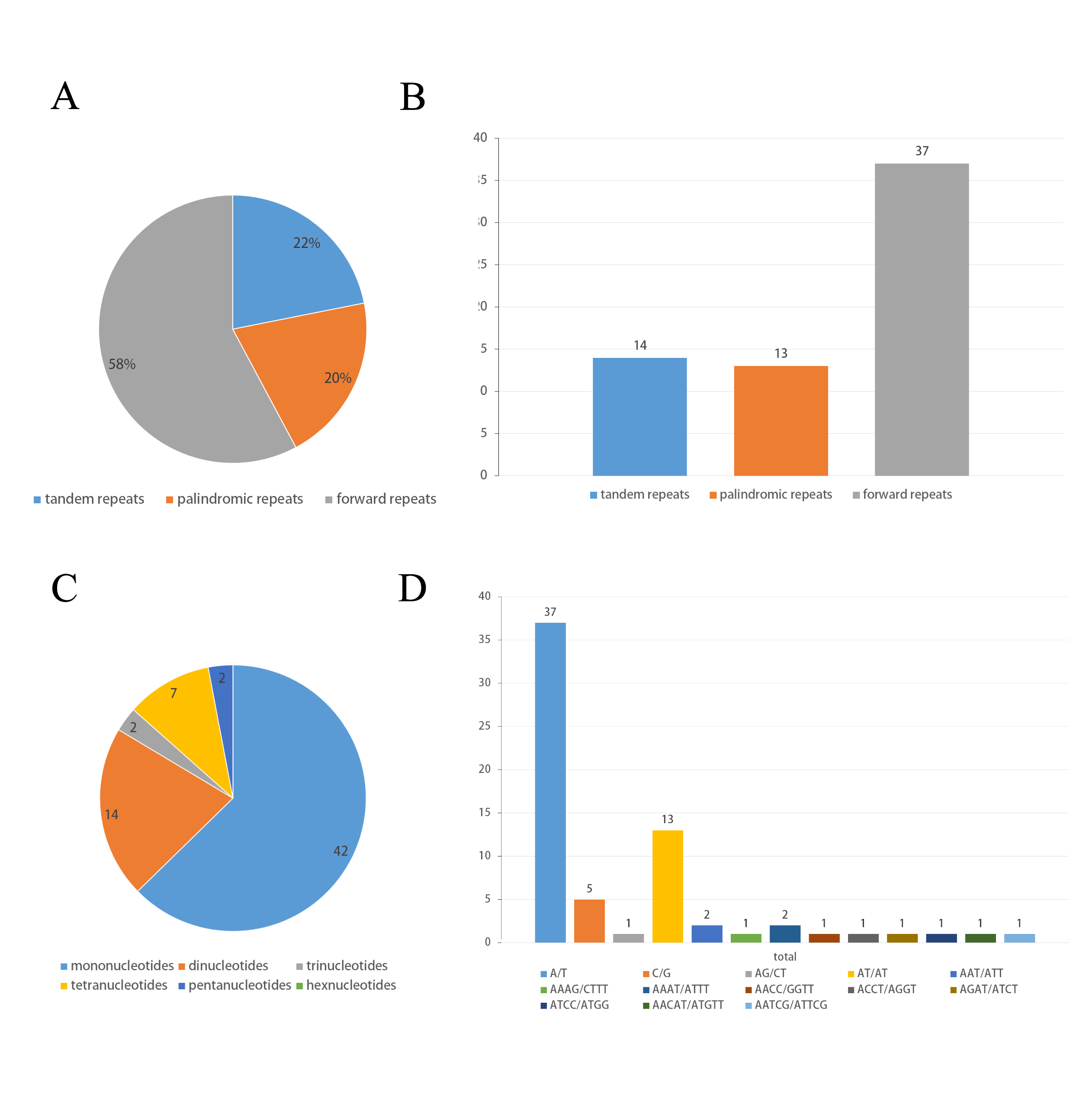
**

**Figure S1**. Types and amount of SSRs (A and B) and long sequence repeats (C and D) in the *Abies ernestii* chloroplast genome.
